# The neural basis of hazard perception differences between novice and experienced drivers - An fMRI study

**DOI:** 10.17179/excli2020-1098

**Published:** 2020-05-04

**Authors:** Seifollah Gharib, Arash Zare-Sadeghi, Seyed Abolfazl Zakerian, Mohsen Reza Haidari

**Affiliations:** 1 Department of Occupational Health, School of Public Health, Tehran University of Medical Sciences, Tehran, I.R. Iran; 2 Finetech in Medicine Research Center, Iran University of Medical Sciences, Tehran, I.R. Iran; 3 Medical Physics Department, Iran University of Medical Sciences, Tehran, I.R. Iran; 4 Section of Neurosciences, Department of Neurology, Faculty of Medicine, Baqiyatallah University of Medical Sciences, Tehran, I.R. Iran

**Keywords:** hazard perception skill, fMRI, novice drivers, experienced drivers

## Abstract

The neural mechanisms underlying hazard perception are poorly understood as to how experience leads to better driving skills. In this study we used functional magnetic resonance imaging (fMRI) to examine experience-related changes in brain activation during hazard perception task between novice and aged drivers. Additionally, region of interest (ROI) and seed-to-voxel analyses were conducted to examine experience-related functional connectivity changes during visual attention and saliency networks between novice (n=15, age 22.13 ± 3.38 years years) and experienced (n=16, age 41.44 ± 5.83 years) drivers. Experienced drivers had significantly lower hazard perception reaction time (1.32 ± 1.09 s) and miss rates (11.42 ± 8.36 %) compared to the novice (3.58 ± 1.45 s and 39.67 ± 15.72 %, respectively). Blood oxygen level dependent (BOLD) activation increased in occipital, parietal and frontal areas when executing hazard perception task in both groups. In general, during the task execution, experienced drivers showed greater activation in the occipital lobe, supramarginal gyrus (SMG), right anterior insular cortex (AIC), anterior cingulate cortex (ACC) and cerebellar regions compared to the novice drivers indicating more efficient visual attention and decision-making process during hazard perception task. Seed based functional analyses during the hazard perception task revealed greater connectivity between the ACC and the entire salience network (visual attention network) in the experienced group. Additionally, ACC had higher functional connectivity with the right frontal eye field (FEF), bilateral intraparietal sulcus (IPS) and lateral occipital areas in the experienced group. Our results suggest that better hazard perception in the experienced drivers is due to increase in the activation of executive attention regions and higher functional connectivity between bilateral occipital cortices and salience network. In conclusion, better hazard perception is highly dependent on emotional awareness, perception of motion velocity, spatial representation of the environment and executing control.

## Introduction

Road accident related deaths are among the leading cause of mortality in recent years with an alarming rise from 1.25 to 1.35 million deaths per year. This rise is more prominent among younger population (WHO, 2015[82], 2018[83]). Accordingly, several attempts have been made to address this issue to the extent that Safer Road Users Program was emphasized as the fourth pillar of Global Plan for the Decade of Action for Road Safety 2011–2020 (Hyder et al., 2017[40]). Hazard Perception Tests or Tasks (HPTs) are routinely employed for distinguishing safe drivers from the careless ones considering the wide range of roads and road users (Grayson and Sexton, 2002[32]; Sagberg and Bjørnskau, 2006[64]; Scialfa et al., 2011[67]; Wetton et al., 2011[80]). It’s thus important to investigate underlying mechanisms of risky driving in young and novice drivers who are presumably more involved in traffic accidents (Ross et al., 2015[61]).

Driving involves continued engagement in a variety of simultaneous tasks including navigation and lane maintenance. Therefore, it requires driver’s full attention in order to drive safely and hence the need to develop and maintain a set of complex perceptual and cognitive skills (Nijboer et al., 2016[52]; Scialfa et al., 2011[67]). 

Safe driving also entails quick and accurate responses to a variety of hazards in the driving environment. Hazard perception is the main component of driving skills and its deficiency is significantly associated with car accidents of considerable consequences (Horswill and McKenna, 2004[39]). Several studies have suggested that Hazard Perception Test (HPT) can differentiate between high-crash-risk group (young novice drivers) and low-crash-risk group of drivers (middle-aged experienced drivers) (Horswill et al., 2008[38]; Sagberg and Bjørnskau, 2006[64]; Scialfa et al., 2013[66]; Vlakveld, 2014[77]). Studies have also reported that HPT can predict crash involvement (Congdon, 1999[17]; Horswill et al., 2015[37]; Pelz and Krupat, 1974[55]) and also plays role in minimizing them (Fisher et al., 2016[29]). However, the underlying neural mechanisms for the differences between young novice drivers and the middle-aged experienced drivers require to be investigated in order to assist the interpretation of HPT results. 

The ability of novice and experienced drivers to identify hazardous traffic events has a reasonable relationship with the collision risk (Fisher et al., 2016[29]). An experienced driver actively looks for information rather than simply reacting to the ongoing events (McKenna and Farrand, 1999[48]). This is related to more passive and inherent pattern-matching cognitive processes. These drivers tend to have a unique set of experiences acquired through their life-long driving career to manage the potential hazards by recalling the previous identical circumstances more effortlessly (Groeger, 2002[33]). Accordingly, proper hazard perception is normally developed based on cognitive resources and entails additional conscious and effortful mental processes that are less likely to become automated. Novice and inexperienced drivers tend to have a distorted visual scanning pattern for perceiving or recognizing the hazards (Chapman et al., 2002[16]; Crundall et al., 2003[22]; Falkmer and Gregersen, 2005[27]).

In addition, hazard perception skills depend not only on sensory information but also on the biases inherent in decision-making processes. There is an important relationship between response-style, risk-taking propensity and hazard perception scores of HPT (Farrand and McKenna, 2001[28]; Rowe and McKenna, 2001[63]). 

Novice drivers have lesser cognitive resource leading to underestimation of hazards, poorer control over the vehicle and faulty decision making (Brown and Groeger, 1988[9]; Ross et al., 2014[62]). Video and computer-based HPTs have shown that novice drivers are deficient in hazard perception skills compared to the experienced ones (Scialfa et al., 2011[67]; Vlakveld, 2014[77]). Neural mechanisms contributing to the superiority of experienced drivers in terms of hazard perception over the novice ones are a more complex process and remain to be investigated.

Hazard perception involves employing several cognitive skills often within a short span of time. Studying its underlying neural mechanisms involves several concepts such as behavioral and environmental prediction, ability to detect hazards (Crundall et al., 2012[23]), situation awareness (Endsley, 1995[26]), signal detection (Parasuraman et al., 2000[54]) and attention (Posner and Petersen, 1990[57]). In particular, situation awareness involves perception of the surrounding environment and comprehension and projection of the future actions within the current situation (Endsley, 1995[26]).

The crucial role and probable relevance of decision-making process to hazard perception also provides a framework to describe and analyze decisions made in uncertain or ambiguous situations (Burge and Chaparro, 2018[11]; Ventsislavova et al., 2016[76]; Wallis and Horswill, 2007[78]). Thus, a multiple-choice HPT can differentiate drivers with various levels of experiences and profiles (offenders and non-offenders) by the application of signal detection theory analyses (Ventsislavova et al., 2016[76]).

It is known that cognitive processes, such as those involved in the hazard perception task are related to activity in different regions of brain. However, few studies have been conducted to objectively investigate their underlying components (Crundall, 2016[21]). Brain imaging techniques can determine the process of hazard perception by identifying the specific regions involved, studying their functions and underlying mechanisms.

Functional MRI currently provides the best non-invasive imaging technique for the localization and evaluation of neural activity. It visualizes human brain structures associated with cerebral oxygenation noninvasively while the individual is performing a cognitive task. This method is based on the fact that oxygenated blood has magnetic properties different from non-oxygenated blood or surrounding tissues (Calhoun, 2008[15]). Blood Oxygen Level Dependent (BOLD) signal for imaging used in fMRI is based on relative levels of oxyhemoglobin and deoxyhemoglobin (oxygenated or deoxygenated blood) contrast and can identify the areas related to cognitive tasks and assess the differences between the involvement of different brain regions and networks (Ogawa et al., 1992[53]).

The objectives of this research were to determine the regions of the brain that are active during the process of hazard perception and differences in the neural activation related to it between the novice and experienced drivers by using fMRI technique. 

As experienced drivers have greater situational awareness, including better sensation and decision-making compared to the novice ones while driving in the hazardous situations, we expected differences in decision making as well as activation and connectivity of intraparietal sulcus (IPS) (Corbetta et al., 2002[18]), frontal eye field (FEF) (Schall, 2004[65]; Mustari et al., 2009[50]), and anterior cingulate cortex (ACC) (Bush et al., 2000[12]; Corbetta et al., 2002[18]) between the two groups as these brain regions are involved in the visual attention, decision making and executive control processes.

## Materials and Methods

### Participants

The participants (all males) for this study were recruited via advertisements and announcements in driving schools and social media. Initially, 81 subjects (39 novice and 42 experienced drivers) agreed to participate after the procedure of the study was explained to them. After the first screening that included driving experience and accident record (novice drivers: less than 6 months period of driving license acquisition and without involvement in any fatal accident; experienced drivers: more than 10 years of driving experience and without any fatal accident and have driving as a daily activity), negative history of neurological and psychological disorders or major systematic illness such as migraine headaches, diabetes, heart disease, head injury, hospitalization for head surgery/illness and absence of non-removable metal in the whole body or metallic tattoos around the head, smoking and addiction to drug/alcohol, 19 novice and 25 experienced drivers were included.

### DASS 21, BIS11 and PSQI screening and clinical examination

After their arrival at the National Brain Mapping Laboratory for fMRI investigation, secondary screening that included depression, anxiety, stress score (DASS 21), Barratt impulsiveness score (BIS 11), Pittsburgh sleep quality index (PSQI) questionnaires and clinical examination was carried out. Considering the effect of depression, anxiety, stress, impulsiveness and sleep quality on visual task performance (including HPT), all the participants were evaluated with validated Persian version of DASS 21, BIS and PSQI written questionnaires (Asghari-Moghadam et al., 2010[2]; Mokri et al., 2008[49]; Nazifi et al., 2014[51]). Those who had high depression, anxiety and stress score (DASS) or high impulsiveness (low BIS) or low sleep quality were excluded from the study. The criteria included in the study were as follows:

- Depression score (from DASS 21) under 9
- Anxiety score (from DASS 21) under 7
- Stress score (from DASS 21) under 14 according to Lovibond study (Lovibond and Lovibond, 1995[45])
- Impulsiveness score (BIS 11) under 72 according to Reid et al. (2014[58])
- Sleep quality score (PSQI) under 5 according to Buysse et al. (1989[13]).

Based on the results of depression, anxiety and stress score (mean ± SD = 9.91 ± 6.18), Barratt impulsiveness index (mean ± SD = 92.66 ± 10.61) and Pittsburgh Sleep Quality Index (mean ± SD = 3.88 ± 1.6, sleep quality = 1, sleep quantity = 6.65 ± 1.4), subjects were found to be in psychologically eligible state. In addition, all the subjects expressed in self-reported questionnaire that they played computer games less than two hours a week. 

Finally, 34 healthy drivers (17 novice, mean age 22.29 ± 3.35, range 19-31 years; and 17 experienced drivers, mean age 42.53, ± 7.22, range 31-52 years), all right-handed, were included in this study for data collection. Two novice and 1 experienced drivers were excluded later from the data analyses because of the artifacts or data recording problems. After drop out, 15 novice drivers (mean age = 22.13 ± 2.38, range 19-27 years) and 16 experienced drivers (mean age = 41.44 ± 5.83, range 33-52 years) were included in fMRI analysis.

None of the participants had any medical restrictions or vision problems for undergoing MRI. Before fMRI, all the participants underwent complete physical and neurological examination. Screening and pre-test clinical examinations were conducted to determine whether the two groups of drivers were healthy and had no medical problems. All were found medically fit to undergo fMRI study.

The study was approved by the IRCCS TUMS Ethics Committee, and all the participants signed written informed consent (Ethics code: IR.TUMS.SPH.REC.1396.4053).

### Procedure

**Figure 1:**
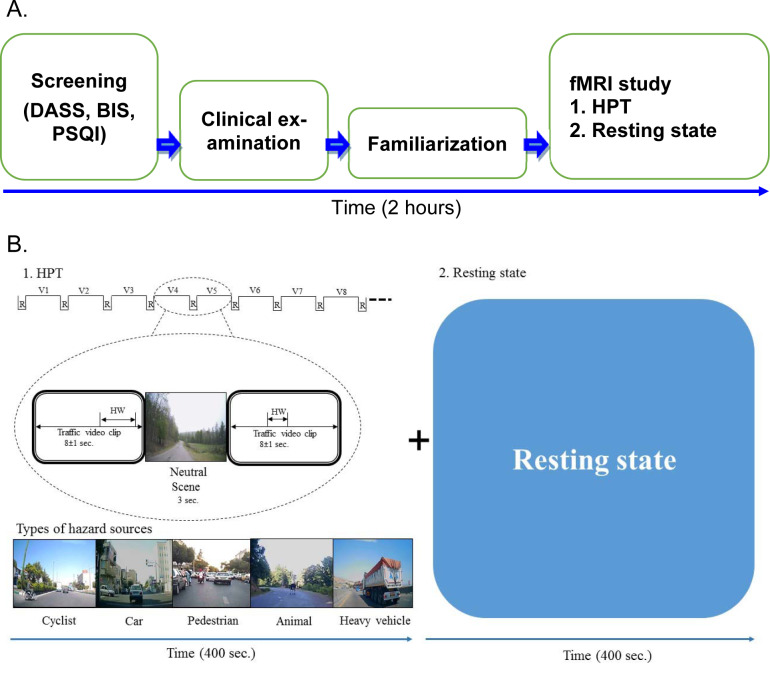
The design of the study A. The overall sequence of the study. B. The design of fMRI study. Thirty-five video clips, each of 8 second duration with different hazard situations were shown to all the subjects. Each video clip had a single hazard source. HW: Hazard window; R: Neutral scene; V: Hazard scene video clip

Figure 1(Fig. 1) (part A) shows the design of the study comprising of 5 sections. After screening tests, written informed consent and clinical examinations, subjects were familiarized with the test procedure at the National Brain Mapping Laboratory, University of Tehran, I.R. Iran. The subjects were verbally informed that in the scanner, clips of various traffic situations (urban, rural, interurban and suburb roads) are going to be shown and that they will see the scene from the front window of the camera car with driver’s view. They were informed of a probable developing hazard in each clip. The subjects were guided to press a button in their right hand whenever they perceive an approaching hazard that needs the driver to respond. They were also told that there were no collisions or questions in the scene. Figure 1(Fig. 1) (part B) shows the fMRI experiment task design. The experiment included 2 sections: Hazard perception task (400 sec.) and resting state (400 sec.). Stimuli were back projected. The watching time and sequence of videos from the beginning to end were same for all the subjects. The HPT was an event design played as a movie without interruptions, and it was same for all the subjects. In the resting state, all the subjects just watched a black screen for 400 sec. The total duration of whole experiment was 2 hours for each subject.

### Hazard Perception Task (HPT) and resting state fMRI

Based on the scenarios leading to accidents with extensive damages that occurred in various (urban, rural, interurban and suburb) locations, hazard perception video clips were prepared from various streets in urban Tehran and its suburbs with the help of police and experts using the validity criteria of Marrington et al. (2008[47]) and Wetton et al. (2010[81]). Out of the total 150 clips, 35 selected clips (8 ± 1 second) were arranged randomly, including hazard situations:

1. Cyclist behavior
2. Car driver behavior
3. Pedestrian behavior
4. Animal on road and
5. Heavy or long vehicles (bus and heavy goods vehicles).

Each clip had a specific (different from others) situation such as scene, hazard window and onset time. Between each hazard situation clip, a neutral scene (3 seconds) of a natural scenery was added. The total duration of this HPT was 400 sec. The subjects were informed before that the HPT (Figure 1(Fig. 1)) would begin with a neutral scene and in between hazard situation clips, the same neutral scene would be repeated signaling the end of clip. Sample video clips SV1 and SV2 are added as supplementary data files. 

After HTP, subjects were given resting state for 400 seconds. During the resting state, a black screen was shown to the subjects. This imaging session was designed to detect differences of default mode network and perform Independent Component Analysis (ICA) between the two groups.

### fMRI (Image Acquisition)

Brain imaging was conducted using 3T MR scanner (Siemens, Prisma model, Iran National Brain Mapping Center, Tehran, Iran) equipped with a 64-channel head coil. Functional images were acquired with T_2_* –weighted echo-planar imaging (EPI); TR = 3 s, TE = 30 ms, flip angle = 90º, matrix 64 _ 64, voxel size = 3 _ 3 mm in-plane, slice thickness = 3 mm; 50 % distance factor; FOV = 448 mm, gap = 0.53 ms, 42 slices, covering the whole cerebral cortex. Resting state data were acquired with T_2_* –weighted EPI (TR = 2500 ms, TE = 30 ms, flip angle = 90º, 42 axial slices, 3.0_3.0_3.0 mm^3^, distance factor = 25 %, FOV = 448_448 mm^2^, interleaved slice ordering). Structural T_1_ – weighted images were collected with a Magnetization-Prepared Rapid Gradient-Echo (MP-RAGE) sequence as an anatomical reference; TR = 2 s, TE = 3.53 ms, TI = 1100 ms, voxel size = 1 × 1 × 1 mm, matrix resolution 256 × 256 × 176, no gap, axial acquisition.

### Data and statistical analyses

#### Participants' characteristics analyses

Participants' statistical analyses were carried out using Statistical Package for the Social Sciences (SPSS) v 16.0. Behavioral variables (stress, anxiety, depression, impulsiveness, sleep quality, miss rates and hazard perception response time) of the two groups (novice and experienced drivers) were analyzed using two-tailed independent sample t-tests. If continuous variables were not normally distributed, or in case of ordinal variables, Mann-Whitney's U test was performed. Miss rates and response time of hazard perception for the five types of hazard sources were analyzed by ANOVA test.

The error condition was defined as no responses, wrong responses, and delayed (after hazard frames disappeared from subject’s view) responses to the hazard situation shown. The level of significance between the two groups was accepted at α = 0.05.

#### Processing and analysis of fMRI data

MRI data preprocessing and analyses (single subject and group analysis) were performed in FSL (FMRIB’s Software Library, version 6.0 http://fsl.fmrib. ox.ac.uk/fsl/fslwiki/FSL) (Jenkinson et al., 2012[42]; Smith et al., 2004[69]). Brain extraction tools (BET) and FMRIB's Linear Image Registration Tool (FLIRT) were performed in FSL (Jenkinson et al., 2012[42]; Popescu et al., 2012[56]). Preprocessing and single subject analyses were performed with fMRI Expert Analysis Tool (FEAT) in the FSL (Jenkinson et al., 2012[42]; Smith et al., 2004[69]; Woolrich et al., 2009[84]). A brain mask from the first volume in the fMRI data was created to get rid of signals outside each subject’s brain (Jenkinson et al., 2012[42]). 

To reduce noise without reducing valid activation, high pass filter with cutoff of 100 s was used, for spatial smoothing full-width at half maximum (FWHM) 5 mm was performed and for slice timing correction, the model of MRI device (Siemens) interleaved was selected. fMRI images were registered to the T1-weighted structural image with the translation (12 degrees of freedom). To correct subjects’ motion and also put all the images into the same space, first we applied 6 degrees of freedom registration on EPI images to transform them into their structural T1 space. Then, we transformed all of them into MNI space using 12 degrees of freedom. The transformation resulted from FLIRT of each subject’s T1 to MNI space.

Group-level fMRI analyses were performed with FEAT and the general linear model (GLM) was used for analyzing response to hazard (Figure 1(Fig. 1)). Error conditions were then gathered and separated as another condition that was ignored. Then, the activation maps for the contrast hazard perception were constructed separately for each subject. Hemodynamic Response Functions (HRF) were modeled by canonical functions at the response time. For each individual, the time courses of responses in different hazard situations based on the time of pressing the response grip (hit rate) were analyzed at the voxel level using a linear regression model. This yielded separate time courses for response to each hazard situation (hit rate) contrasted with the time courses for missing of hazard and natural scene (without hit rate). The contrast images of every participant from the first level were subjected to a second-level of analysis using two sample t-test to see differences in contrast between the two groups.

#### Functional connectivity analysis of HPT

The task fMRI scans were analyzed using CONN functional connectivity toolbox version 18b to create individual and group functional connectivity maps (Gabrieli Lab, Massachusetts, USA). Data of the both groups of subjects were preprocessed to the 6 motion correction parameters and their first temporal derivatives, global grey matter (GM), white matter (WM), and cerebrospinal fluid (CSF) were included to remove variance related to motion, the global GM, WM, and CSF signals, respectively. Realignment parameters, WM and CSF were entered as confounds in a first-level analysis and the data were band-pass filtered to 0.01 Hz.

To examine functional connectivity involved in the task, the seed region of interest (ROI) consisting of 6 mm radius spheres centered on MNI coordinates were used to identify the corresponding visual saliency and attention networks as follows: Dorsal Attention Networks (left and right IPS: -39-4352/ 39-4254, left and right FEF: -27-964 / 30-664); Salience networks (ACC: 0 22 35, left and right Anterior Insula: -44131 / 47140, left and right Rostral Prefrontal Cortex (RPFC): -324527 / 324627, left and right Supramarginal Gyrus (SMG): -60-3931 / 62-3532); Visual Networks (Occipital: 0-93-4, Medial: 2 -79 12, Left and right lateral: -37-7910 / 38-7913) were selected.

The mean time series from each ROI was used as a predictor in a multiple regression general linear model (GLM) at each voxel. Then, an ROI analysis and a seed-to-voxel analysis were conducted. The ROI-to-ROI analysis was used to test hypotheses regarding the functional connectivity differences between the two groups in visual attention and saliency networks. To show the regions that significantly correlated with the seed ROI, the CONN generated for each network with threshold at the whole-brain cluster-level corrected alpha was considered at 0.05, and for the voxel-wise detection of significant differences between the groups, p-value of 0.001 was selected.

#### Functional connectivity analysis of resting state

While in the resting state, the seed ROIs consisting of 6 mm radius spheres centered on MNI coordinates were used to identify the corresponding Default Mode Networks as follows: PCC: 1 -61 38, MPFC: 1 55 -3, left and right LP: -39-7733/ 47-6729.

In addition, for the resting state data analysis, we used standard MELODIC tool of FSL, which is dedicated to the resting state fMRI analysis based on Independent Component Analysis (ICA). For the preprocessing steps, each functional volume was registered to the T1 space and then, using the subject’s T1 to MNI space registration transform, all of them were normalized to the MNI standard space. They were then corrected for the motion artifact and slice timing and were finally smoothed with a 5 mm kernel. 

## Results

Table 1(Tab. 1) shows the differences in the characteristics of the two groups, i.e., the novice and the experienced drivers considering the years of education, DASS, impulsiveness, sleep quality, years of driving experience, average kilometers of driving distance and age. There were significant differences (p < 0.001) between age, driving experience in years and average kilometers of driving distance (km/week) of the two groups (Table 1(Tab. 1)). 

There are significant differences in age, driving license acquisition, driving experience and average of driving distance (km/week) between the two groups. Given the nature of the groups studied, these differences were expected. Key: DASS, Depression Anxiety Stress Score; BIS, Barratt Impulsiveness Scale; PSQI, Pittsburgh Sleep Quality Index (lower score is higher quality of sleep in last month). A 4-point Likert scale was used for sleep quality values between 0 (high) and 3 (poor) quality of sleep in the last night. *Mann-Whitney test, other p-values were analyzed by student’s t test. 

### Behavioral performance result

Results of ANOVA for the overall performance (mean Hazard Perception Reaction Times (HPRT) and mean Hazard Perception Miss Rates (HPMR)) in both the groups revealed no significant differences between driving situations with different hazard sources. For novice drivers: F_ HPRT_ = 1.78, p _HPRT _= 0.16, F_ miss rate_ = 0.54, p _miss rate_ = 0.70 and degrees of freedom = 4, 30. For experienced drivers: F_ HPRT_ = 0.72, p_ HPRT _= 0.58, F_ miss rate_ = 0.50, p _miss rate_ = 0.73 and degrees of freedom = 4, 30. However, the overall performance of the experienced drivers compared to the novice ones in the hazard perception task was better.

### Hazard Perception Reaction Times (HPRT)

The mean HPRT during the HPT in the MRI scanner were 3.58 ± 1.45 sec. and 1.32 ± 1.09 sec. for the novice drivers and experienced ones respectively. The t-test revealed significant effects of experience in HPRT. Significant difference between the two groups in HPRT (t_2, 68_ = 7.35, p < 0.001) showed that novice drivers had significantly longer HPRT compared to the experienced ones in all hazardous situations. Table 2(Tab. 2) shows the results of HPRT in the different hazard sources for the novice and experienced drivers. The results of ANOVA for the reaction times in both groups revealed no significant differences between different hazard sources (p > 0.05).

### Hazard Perception Miss Rates (HPMR)

Table 2(Tab. 2) summarizes the miss rates of the two groups. The mean HPMR for the novice drivers were 39.67 ± 15.72 %, and 11.42 ± 8.36 % for the experienced drivers. The t-test for mean HPMR in the HPT that was performed inside the MRI topographer revealed a significant difference between the novice and the experienced drivers in all situations of road hazards (t_2, 68_=8.99, p <0.001). Mean miss rate of HPT for the novice drivers was significantly (28.25 %) higher than that of the experienced drivers. In addition, the ANOVA for the miss rates in both the groups revealed no significant difference between different hazard sources (p > 0.05).

### fMRI task results

**Figure 2:**
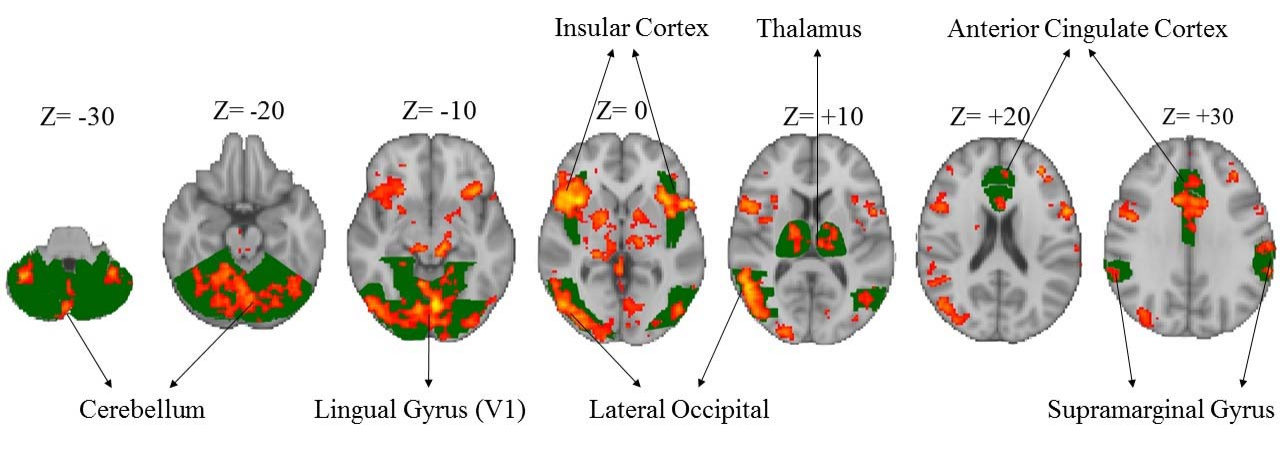
Significant activation of different areas of the brain associated with hazard perception task in both the groups. Green colors show the specific brain areas and red/yellow colors indicate activated areas of brain.

The HPT revealed significant activation of different areas of the brain (p< 0.05, corrected from multiple comparisons) associated with hazard perception [hit rate – without hit rate (including missing of hazard and neutral scene)] (Figure 2(Fig. 2)), including lateral occipital (x = 54, y = -56, z = 8); lingual gyrus (x = -2, y = -72, z = -10); insular cortex (x = 36, y =16, z = 0); FEF (x = 44, y = 2, z = 40) and cerebellum (x = -4, y = -72, z = -20) in all the subjects. This task was then examined in more detail by considering the mean activation within each group separately. In the majority of these areas, the results demonstrated higher brain activation in the experienced drivers compared to the novice ones.

### Comparison of BOLD-signal between the two groups in HPT

**Figure 3:**
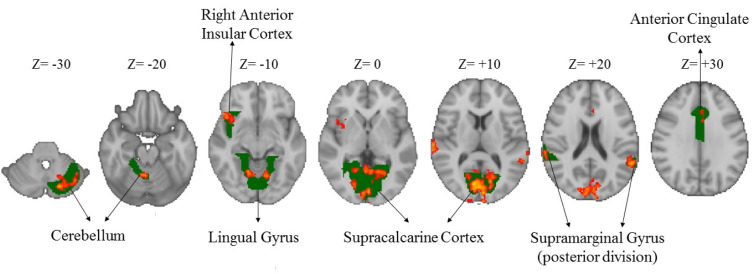
Greater brain activation in the experienced drivers compared to the novice ones during the HPT (Z-scores). Green colors show the specific brain areas and red/yellow colors indicate activated areas of brain.

Compared to the novice drivers, the experienced drivers showed greater brain activation during HPT in five areas. Figure 3(Fig. 3) shows the brain areas with significantly increased neural activity associated with HPT in the experienced group compared to the novice ones (red/yellow blobs) on MNI template. These included the occipital lobe (supracalcarine cortex (x = 0, y = -80, z = 8; Z = 4.68), lingual gyrus (x = 14, y = -58, z = -8; Z = 4.28)), bilateral SMG (x = -58, y = -48, z = 20; Z = 3.99 and x = +62, y = -42, z = 12; Z = 4.51), right anterior insular cortex (AIC) (x= 40, y= 12, z= -4; Z= 4.14), FEF (x= 0, y= 16, z= 46; Z= 3.82), ACC (x=0, y=32, z = 24; Z = 3.12) and cerebellum (x = -18, y = -76, z = -32; Z = 4) as shown in Table 3(Tab. 3).

### Task functional connectivity analyses

The first ROI-to-ROI analysis was performed to investigate the brain regions that were engaged in hazard perception performance (visual, dorsal attention, salience networks of the brain) in the novice and experienced drivers. There were significant connectivity differences in the salience networks between the novice and experienced drivers. 

**Figure 4:**
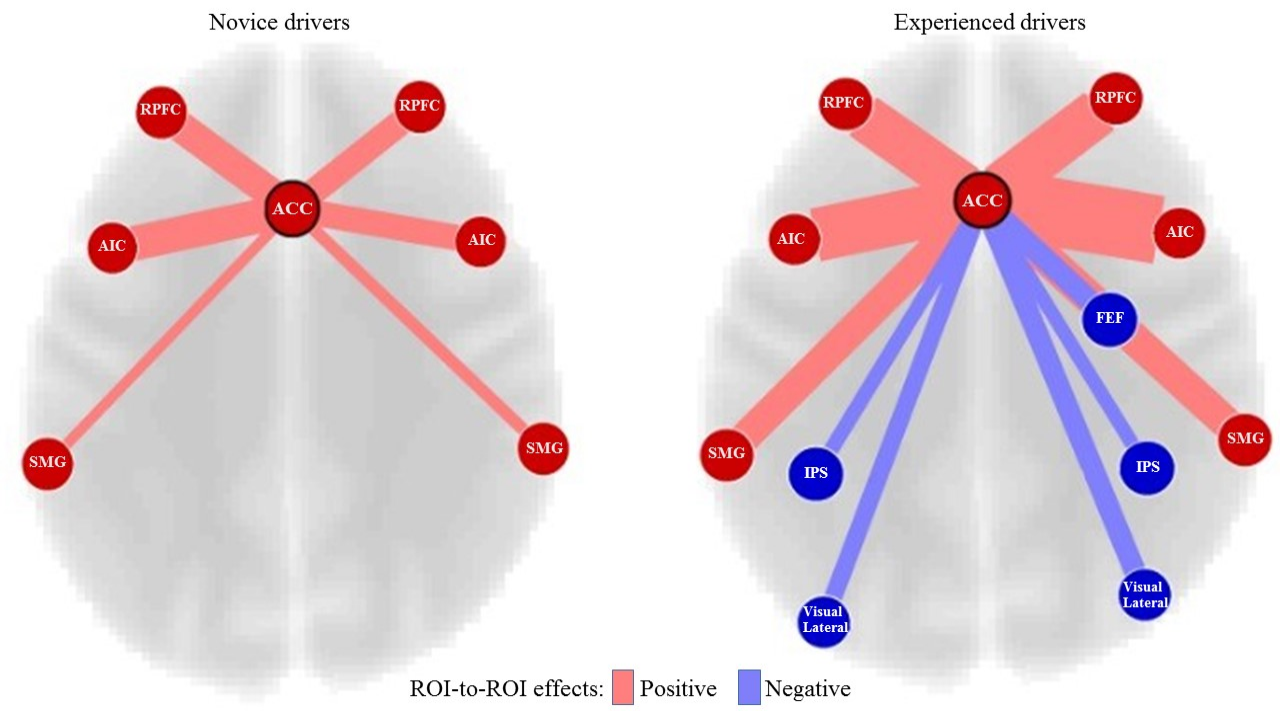
Connectivity differences of ACC between the novice and experienced drivers. Key: ACC, Anterior Cingulate Cortex; RPFC, Rostral Prefrontal Cortex; AIC, Anterior Insular Cortex; SMG, Supramarginal Gyrus; FEF, Frontal Eye Field; IPS, Intraparietal Sulcus

The most significant connectivity differences between the two groups were related to the ACC (t = 9.34, p < 0.001). Figure 4(Fig. 4) shows the ACC connectivity with different regions in the two groups of drivers. The experienced drivers showed significantly stronger (positive) connectivity between ACC and right AIC (t = 5.24, p = 0.002) and between ACC and bilateral SMG (ACC-right SMG (t = 2.46; p = 0.041) and ACC-left SMG (t = 2.66; p = 0.03)). However, the experienced drivers did not show greater connectivity between ACC and bilateral RPFC or left anterior insula at the level of significance (i.e., p < 0.05, FDR corrected).

### Seed-to-voxel analysis

**Figure 5:**
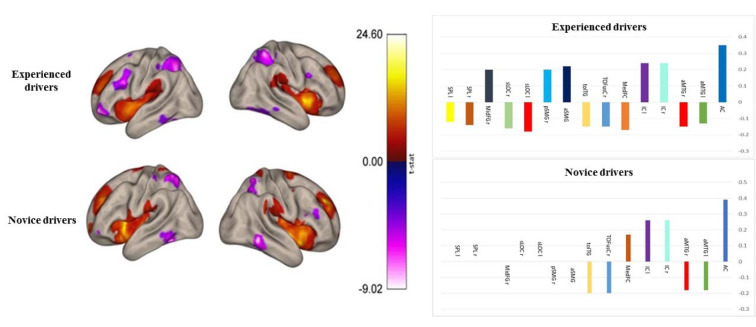
Group contrasts of seed-to-voxel connectivity maps with ACC seed showing increased functional connectivity associated with greater right frontal pole and right lateral occipital cortex, superior division in the experienced drivers. Key: AC, Anterior Cingulate; aMTG l, anterior division of Middle Temporal Gyrus left; aMTG r, anterior division of Middle Temporal Gyrus right; IC r, Insular Cortex right; IC l, Insular Cortex left; MedFC, Frontal Medial Cortex; TOFusC r, Temporal Occipital Fusiform Cortex right; toITG l, Inferior Temporal Gyrus, temporooccipital part left; aSMG l, Supramarginal Gyrus, anterior division left; pSMG r, Supramarginal Gyrus, posterior division right; sLOC, Lateral Occipital Cortex, superior division left; sLOC r, Lateral Occipital Cortex, superior division right; MidFG r, Middle Frontal Gyrus right; SPL r, Superior Parietal Lobule right; SPL l, Superior Parietal Lobule left

ROI-to-ROI analysis showed functional connectivity differences between the two groups in ACC and the regions of the salience network. Therefore, we carried out seed-to-voxel analysis to investigate whether there was greater connectivity with the regions outside the networks. Whole-brain between group seed-to-voxel analysis was performed to examine the potential group differences in the functional connectivity between ACC and all other voxels in the brain. The experienced group showed stronger (negative) connectivity between ACC and a large cluster of voxels in the IPS, bilateral occipital cortices and right FEF, as seen in Figure 4(Fig. 4). Thus, compared to the novice drivers, the experienced drivers showed different patterns of connectivity between the ACC and regions of salience network, dorsal attention network and vision. Figure 5(Fig. 5) shows the final group contrasts of seed-to-voxel connectivity maps with ACC seed within different clusters.

### fMRI resting state result

ROI-to-ROI analysis of resting state was performed in the default mode regions to investigate passive brain network in the two groups. This analysis showed that there was no significant difference between the two groups in the connectivity of default mode regions (t = -0.268, p = 0.789). Resting state analyses were performed to verify that the brains of the two groups of drivers are normal and healthy, and that there was no significant difference between them without any stimuli or tasks.

### Independent Components Analyses (ICA)

After the first level of group analysis (gICA Results), thirty independent components were found based on the defined contrast. To find the components that were significantly different between these two groups, we applied multiple comparison and dual regression on the results of the first level group analysis. The outcome of dual regression analysis showed that there was no component producing significant differences between the two groups. In addition, the independent components didn't pass the dual regression for multiple comparison correction indicating that there was no significant difference in the resting state of brain between the two groups.

## Discussion

The present study used fMRI to detect functional brain regions and their connectivity for understanding the risk of driving and their differences between the novice and experienced drivers. The images of active brain regions and functional connectivity of participants in HPT - where they responded to the usual driving hazards on different roads (for example, when a pedestrian or a cyclist unexpectedly interrupted their way), were compared between the novice and experienced drivers. It also provided the fMRI-based evidence for the psychological studies on hazard perception such as situational awareness and the effect of attention networks on driving performance.

In summary, this study developed a non-invasive method to detect the functional differences during driving HPT and evaluated the brain regions and neural networks involved in it. It also revealed behavioral differences in the performance and functional connectivity between novice and experienced drivers. Our results showed that in experienced drivers, the activity of brain regions associated with spatial representations of the environment, perception of motion velocity, attentional orientation and executing control was significantly higher than that of novice drivers. Analysis of the functional connectivity during HPT showed that the relevant attention and focus networks were different between the two groups of drivers. 

### Hazard perception and the brain activation

Overall, study results indicated higher level of neural activity in the occipital lobe, including lingual gyrus, middle temporal, and lateral occipital cortices, that are related to spatial awareness, perception of distances between environmental elements and motion analysis while performing HPT compared to the other areas of the brain (Figure 2(Fig. 2)).

Traffic information includes awareness about the speed of driver’s own and the surrounding vehicles, the position of driver’s own vehicle, the detection of immediate future hazards (for example, pedestrians, other vehicles and obstacles), and perception of surrounding environment, etc. (Benedikt, 2011[4]). The present study reinforces the role of the neural components of the visual system in safe driving and hazard perception. Integrity of this system has been one of the most important elements for obtaining a license and driving. Our results suggest that neural activity in most of the brain areas related to spatial perception and movement is pivotal for the hazard perception. For example, FEF had significant activity in driving risk perception as it plays an important role in attention, focus, eye movements, and top-down sensory processing in brain's rapid response to external stimuli. Neuronal activation in the FEF leads to enhanced activity of occipital lobe while detecting hazard (Mustari et al., 2009[50]; Schall, 2004[65]). Gaze direction of the eye pupil measured using an eye tracker has been used as parameter for measuring the ability to sense hazard (Caird et al., 2008[14]; Crundall et al., 2004[24]; Land, 2006[43]). However, recently it is suggested that the gaze direction doesn’t mean paying attention and perception of the hazard of the object or subject (Fisher et al., 2016[29]). In addition, different brain areas are activated in perception of driving hazard and are not solely dependent on the activity of the FEF as measured by the eye tracker.

Insular cortex plays an important role in driving hazard management. Brain imaging studies have suggested that its function is related to emotional awareness (Craig and Craig, 2009[20]), interoceptive awareness (Zaki et al., 2012[86]), risk prediction and uncertainty estimation (Bach and Dolan, 2012[3]; Bossaerts, 2010[7]). Insular cortex is also necessary for bottom-up and top down integration and emotional awareness in diverse cognitive tasks (Gu et al., 2013[34]). Thus, it plays a key role in emotional awareness related to a traffic event and contributes to the cognitive control and reaction to the hazardous situations. Activation of insula increases attention necessary for avoiding collisions with approaching objects (Spiers and Maguire, 2007[70]).

Findings of this study showed that parts of the cerebellum play a significant role in the perception of driving hazard. As the primary function of the cerebellum is to coordinate movements of the body and eyes, (Buckner, 2013[10]) this activity may be due to the pupillary movement and visual feedback of the drivers participating in the study while viewing the clips on the scanner (Uchiyama et al., 2003[72]). Activation of cerebellum in cognitive and emotional tasks is reported previously (Adamaszek et al., 2017[1]; Buckner, 2013[10]; Strick et al., 2009[71]), which is consistent with the results of this study.

Overall, our results suggest that situational and emotional awareness are necessary for hazard perception and decision-making in a hazardous situation. In addition, driver’s decision-making based on hazard perception is not only related to approximate information received from images such as motion and cues and prior exposure to the road hazards, which represents a summary of hazardous situations in one's mind. Significant activities of the thalamus, IPS and prefrontal cortex in drivers of this study suggest that the hazard perception skills are also related to the brain regions associated with different attention networks, i.e., alerting, orienting and executive control.

### Greater brain activation in experienced than novice drivers

Experienced drivers with better driving hazard perception (both in percentage of correct responses and in reaction time) compared to novices, had significantly greater brain activity in the following regions (Table 3(Tab. 3), Figure 3(Fig. 3)):

- Lingual gyrus: It has the V1 (ventral stream) concentrated and is related to awareness about the object and the features of what is being observed (Goel and Dolan, 2001[31]).
- Supracalcarine cortex: It is part of the dorsal stream region associated with spatial representation and location awareness of what is seen (Borra and Rockland, 2011[6]).
- Supramarginal gyrus: It plays role in attention to the location, objects, individuals and prediction of posture and behavior (van Harskamp et al., 2002[75]).
- Anterior insular cortex: It plays important role in emotional awareness (Craig and Craig, 2009[20]).
- Anterior cingulate cortex: It plays a critical role in attention and helps to decry effects of distraction agents in executive planning and control (Bush et al., 2000[12]).

As reported previously, experience is an important factor in identifying hazardous situations as it provides better abstract representation (Crundall, 2016[21]; Horswill and McKenna, 2004[39]; Scialfa et al., 2013[66]). Findings of our study have also revealed significant effects of experience on driving hazard perception and it elaborates the role of experience as follows:

1. In this study, activity of the occipital lobe was significantly higher in experienced compared to inexperienced drivers, indicating that driving experience enhances the skill of having a detailed map of the visual field and the spatial relationships between environmental elements. This skill also requires top-down attention and more pupillary movement, causing higher activity of cerebellum.

In the experienced drivers, the dorsal stream which plays role in spatial representation is strongly connected with FEF and prefrontal cortex regions that are involved in signal detection. This can be a reason for the faster decision-making process during hazard perception among the experienced drivers. Studies using an eye tracker have also shown that focusing on the hazard and pupillary movements of the experienced drivers were significantly greater than inexperienced drivers (Falkmer and Gregersen, 2005[27]).

1. Our results showed that areas associated with motion perception and behavior prediction were more active in experienced drivers, indicating that one of the important inputs in driving hazard perception is the proper assessment of movement. Distance of an approaching hazard from the vehicle greatly influences the driver's decision making. Accurate estimation of distance and speed is crucial for hazard perception skills. There is a significant relationship between speed and hazard perception (Crundall et al., 2012[23]; Horswill and McKenna, 2004[39]; Renge, 1998[59]). Speed is an influential dimension in responding to road hazards in the HPT (Scialfa et al., 2013[66]). Results of studies on risk perception with what-happen-next approach (Coyne et al., 2007[19]; Lim et al., 2014[44]; Ventsislavova et al., 2016[76]) are consistent with our results suggesting that understanding movement and predicting the near future can be the two very important inputs for deciding how to respond to road hazards. These findings apparently contradict Groeger's (2002[33]) proposed mechanism, suggesting that the experienced drivers in a process without effort and cues recovered from their memory, have better performance in hazard perception.
2. Results of our study showed higher situational awareness as the reason for identifying a specific hazard more accurately and with higher hit rate in the experienced drivers. These are consistent with the findings of previous studies (Horswill and McKenna, 2004[39]; Underwood et al., 2013[73]; Vlakveld, 2014[77]) and provide evidence based on fMRI.
3. Experienced drivers in our study made faster decisions in identifying hazardous situations (shorter duration of response to stimuli) (Table 2(Tab. 2)), which can be attributed to the activity of prefrontal cortex (ACC and AIC). Experienced drivers make faster decisions in hazardous situations because of their higher situational awareness and sensitivity to hazardous conditions and lower risk acceptance thresholds (Benekos and Diamantidis, 2017[5]; Deery, 1999[25]). The response speed also depends on the degree of adaptation of the proximal stimulus and one's "mental model" of hazards (Scialfa et al., 2013[66]).

The AIC plays important role in the emotional awareness and consequence-attention-based decision making (Craig and Craig, 2009[20]; Gu et al., 2013[34]; Zaki et al., 2012[86]), which can lead to faster response speed of experienced drivers in the presence of hazard stimuli in HPT. Perhaps the reason for the significant difference in cerebellar activity between the experienced and novice drivers is the difference in emotional affect resulting from driving images as a driver (Horikawa et al., 2005[36]; Spiers and Maguire, 2007[70]). Age-related alterations in amygdala and cerebellar connectivity from young to middle adulthood on emotion regulation (Xiao et al., 2018[85]) can also explain the results of our study. In addition, behavioral and environmental prediction is reported to be less developed in novice drivers (Vlakveld, 2014[77]). Overall, the findings of our study suggest that experienced drivers with greater brain effort, emotional awareness and cognitive activity have better performance in hazard perception compared to the novice ones. 

Novice drivers devote considerable part of their cognitive resources to the vehicle control compared to the visual search by the experienced ones. This results in lower hazard perception and lesser allocation of cognitive resources among the novice drivers that is observed in performing the HPT (Boufous et al., 2011[8]; Brown and Groeger, 1988[9]; Nijboer et al., 2016[52]; Ross et al., 2014[62]).

Results of the present study showed that upper parts of the ACC had more activation in decision-making in response to hazard. These regions are activated in rational cognitive responses (Uchiyama et al., 2003[72]) and ACC activation helps to decrease the effect of distracting factors on executive function (Bush et al., 2000[12]; Spiers and Maguire, 2007[70]). Therefore, responding to the driving hazards is more of a rational cognitive response. 

### Experience-related differences in the brain attention networks on hazard perception

The findings of the present study showed that the connectivity of brain regions related to the executive control attention network (in ACC) differed between the two groups during HPT. As ACC has been found to be highly connected with visual areas of the brain related to perception of space, movement and visual search in experienced drivers (Figure 4(Fig. 4)) (Janzen and Van Turennout, 2004[41]; Malach et al., 1995[46]), it indicates the differences in the mechanism of hazard perception in dividing and focusing attention between the two groups of drivers. The executive control attention network plays an important role in safe driving (Roca et al., 2013[60]; Underwood, 2007[73]) as there is a significant relationship between the occurrence of a driving collision and the executive control attention function (Roca et al., 2013[60]). However, no relationship was previously found between the attention functions and driving performance (Weaver et al., 2009[79]). Our results indicate that the activity of attention-related regions in experienced drivers was significantly higher than novice drivers. In addition, the attention network of the executive control was different between them. This difference in activation of ACC could be due to the negative connectivity with the regions of motion perception and the speed assessment in experienced drivers (Uchiyama et al., 2003[72]). Delay in braking during hazardous situations is attributed to the executive control network (Roca et al., 2013[60]). Based on the results of the present study, the delay in novice drivers can be related to the impact of the negative relationship between ACC and lateral occipital cortices on decision-making (Hahn et al., 2006[35]).

### Similarities of brain behavior of the drivers at resting state (without task)

Findings of ICA and DMN analyses of the resting (non-tasked) state of the drivers showed that there were no significant differences in the activities of brain regions and networks between the two groups. In addition, the resting state analysis of the brain showed no difference between the brains of novice and experienced drivers. In addition, their brain activity in the resting state had no superiority over each other. As this study was conducted in a country where hazard perception training is not provided for drivers at any level, and all the participants had no training in this regard. Therefore, the differences found in this study are due to the effect of driving experience.

## Conclusions and Limitations

The present study emphasizes the differences in neural activity between the novice and experienced drivers during hazard perception task and based on fMRI, found objective evidences that explain differences in driving behavior of the two groups of drivers. Our study is just the first step to introduce a rather expensive alternative based on brain imaging tools for the current methods used in HPT that are mostly behavioral. This alternative can be used to provide and extend a certificate for people with certain conditions such as probability of injury in the accidents, being on psychiatric medications, or driving after surgery, etc.

Subsequent researches could answer the following questions: Could this technique quantify the neural differences before and after hazard perception training in young people? Could this method make a significant difference between the trained and untrained drivers? Further studies could also contribute to the development of other brain imaging methods. These methods are somewhat expensive for research; however, if they achieve a reliable design and can be used in the certification process, they can provide more reliable data on the mental health of drivers which is the most important part of the transport fleet. Application of the findings of this study from another viewpoint could change the approach of hazard perception training towards attention-based training and greater spatial perception and perceived risk of road hazards. The results of future studies in this area can be helpful.

Due to the difficulty of finding particularly experienced female drivers who could be willing to participate in the study, it was not possible to conduct a female gender test. According to previous studies (Glendon et al., 1996[30]; Scialfa et al., 2011[67]; Scrimgeour et al., 2011[68]; Ventsislavova et al., 2016[76]), there are no significant gender-based differences in the risk perception task. However, there may be some differences in neural functions in the HPT that make it necessary to conduct further researches in this regard.

## Acknowledgements

Authors would like to thank the participants in this study. Thanks to Willem P. Vlakveld for his assistance. This work was financially supported by a grant from the deputy of the research review board and the ethics community of the Tehran University of Medical Sciences (Grant NO: 38437-27-02-97) and carried out at the National Brain Mapping Laboratory of Iran.

## Conflict of interest

The authors declare no conflict of interest. 

## Supplementary Material

Supplementary material 1

Supplementary material 2

## Figures and Tables

**Table 1:**
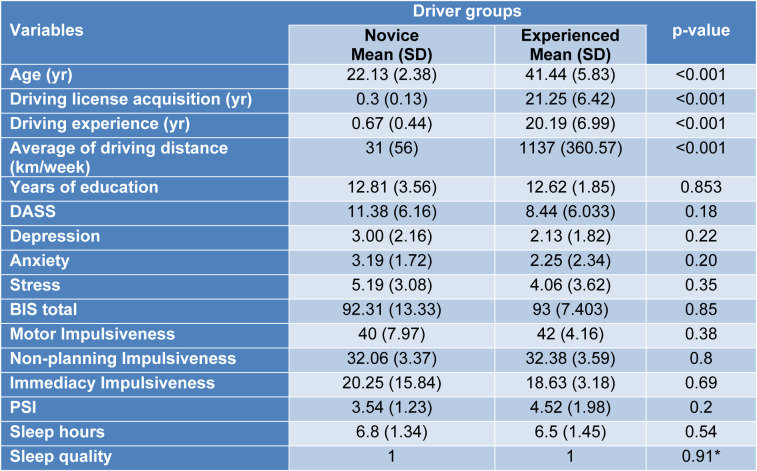
Demographics and test variables of participants

**Table 2:**
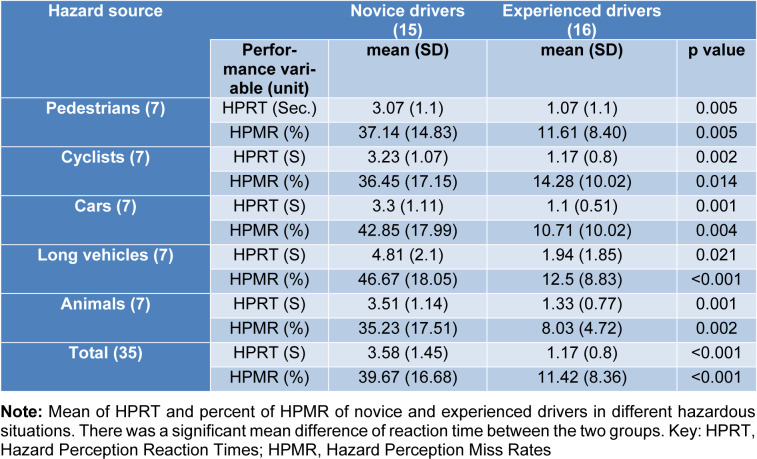
Hazard perception performance of drivers in different kinds of hazardous situations

**Table 3:**
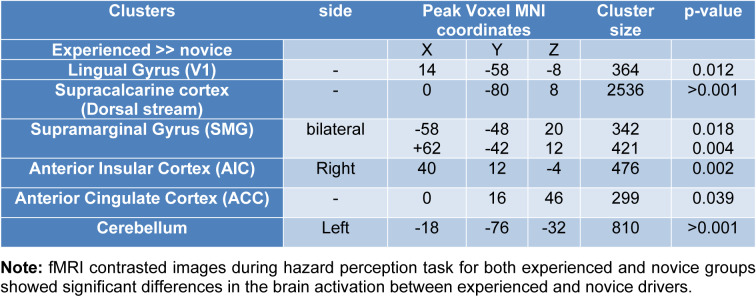
Differences between novice and experienced drivers on BOLD-signal during HPT
